# Sleep and Caregiver Burden Among Caregivers of Persons Living With Dementia: A Scoping Review

**DOI:** 10.1093/geroni/igae005

**Published:** 2024-02-14

**Authors:** Meghan K Mattos, Veronica Bernacchi, Kelly M Shaffer, Virginia Gallagher, Shinae Seo, Laura Jepson, Carol Manning

**Affiliations:** Acute and Specialty Care Department, School of Nursing, University of Virginia, Charlottesville, Virginia, USA; Division of Geriatrics, School of Medicine, University of Virginia, Charlottesville, Virginia, USA; College of Nursing, Michigan State University, East Lansing, Michigan, USA; Center for Behavioral Health and Technology, School of Medicine, University of Virginia, Charlottesville, Virginia, USA; Department of Neurology, School of Medicine, University of Virginia, Charlottesville, Virginia, USA; Acute and Specialty Care Department, School of Nursing, University of Virginia, Charlottesville, Virginia, USA; Acute and Specialty Care Department, School of Nursing, University of Virginia, Charlottesville, Virginia, USA; Department of Neurology, School of Medicine, University of Virginia, Charlottesville, Virginia, USA

**Keywords:** Alzheimer’s disease, Informal caregiver, Sleep quality

## Abstract

**Background and Objectives:**

Caregivers of persons with dementia report worse sleep when compared to the general population. The objective of this review was to synthesize evidence regarding the link between caregiver burden and dementia caregivers’ sleep.

**Research Design and Methods:**

We conducted a scoping review using a systematic search for pertinent literature in PubMed, CINAHL, and Web of Science through March 2022. Keywords included content areas of dementia, caregiver burden, and sleep. Inclusion criteria were informal caregivers of persons living with dementia, a measured relationship between informal dementia caregiver sleep and subjective caregiver burden variables, and original research. Non-English studies were excluded. Extracted data were organized in tables, compared, and synthesized.

**Results:**

The search yielded 540 nonduplicate articles screened by title and abstract; 118 full-text articles were reviewed; 24 were included. Most studies were cross-sectional, with variable sample sizes. Dementia caregivers had significantly poorer overall perceived sleep than noncaregivers across 4 studies that examined self-reported sleep measures. Eighteen studies investigated the association between caregiver burden and self-reported sleep quality, with 14 reporting a significant positive association between caregiver burden and self-reported sleep quality, and 4 finding null results. Only 2 of the 4 studies reporting the association between caregiver burden and objective sleep parameters (ie, actigraphy and polysomnography) reported a significant positive association for at least one sleep subdomain.

**Discussion and Implications:**

Although subjective sleep quality is commonly affected by dementia caregiving burden, there is a lack of corresponding evidence on the relationship between burden and objective sleep metrics. Healthcare providers should consider the dementia caregiver burden’s impact on sleep and regularly assess caregivers’ sleep difficulties. Future studies should focus on consistently measuring caregiver burden and sleep to promote dementia caregiver health and well-being.

**Translational Significance:** Although sleep problems are commonly reported among dementia caregivers, the nature and frequency of caregiver sleep disruptions and their relationship to health status have not been systematically mapped. This review aimed to synthesize evidence regarding the link between caregiver burden and dementia caregivers’ sleep. Caregivers of persons living with dementia were found to have poorer self-reported sleep quality compared to their age-matched counterparts. Although self-reported overall sleep quality is associated with dementia caregiving burden, there is mixed evidence on the relationship between sleep subdomains and burden. Further research examining this relationship is critical to improving the health of dementia caregivers.

Dementia is a syndrome that leads to the deterioration of cognitive abilities and may affect one’s ability to perform activities of daily living. The cognitive changes not only interfere with the daily life of the person living with dementia but also affect families, friends, and communities. Whether it be family members or friends, an estimated 11 million U.S. adults provide unpaid care for dementia patients (1), and the significant deleterious effects of caregiver burden on health outcomes of caregivers and care recipients are a significant public health concern (2,3). Caregiver burden is defined as “the strain or load borne by a person who cares for a chronically ill, disabled, or elderly family member” (4). Caregivers are often referred to as “invisible second patients” due to the high rates of burden, social isolation, and financial hardship that influence their mental and physical health (5).

One aspect of a caregiver’s daily life that affects dementia caregiver burden is sleep. It is known that the nighttime behaviors of people with dementia contribute to caregiver sleep disruption (6) and may lead dementia caregivers to experience poorer sleep quality and higher rates of sleep disturbances compared to the general population. Specifically, up to 67% of dementia caregivers experience sleep disturbances compared with up to 50% of the general population (7). Worse sleep efficiency, the percentage of time asleep while in bed, is known to predict worse mental health in caregivers (8). There are bidirectional associations between mood disorders such as depression and sleep disturbances (7,9,10). Sleep impairment contributes to caregivers’ anxiety and physical morbidity, affecting caregivers more severely than the general population (7,11,12). Moreover, caregivers’ poor sleep affects their care recipients, which is further compounded by care recipients’ sleep disturbances (13–16).

A recent meta-analysis examining caregivers of adults with Alzheimer’s disease (AD) found that caregivers experienced fewer hours of sleep and worse sleep efficiency than their noncaregiving counterparts (17). Although the amount of time someone sleeps directly affects health, sleep quality is equally important and can influence mood, memory, decision making, judgment, and one’s ability to learn new things. The meta-analysis revealed that sleep quality was most commonly assessed using the self-report Pittsburgh Sleep Quality Index (PSQI), which has 7 subscores: subjective sleep quality, sleep latency, sleep duration, habitual sleep efficiency, sleep disturbances, use of sleep medication, and daytime dysfunction. The meta-analysis provided significant evidence that caregivers of persons with AD report worse quality sleep; however, the factors contributing toward worse sleep quality and potential modifiable factors to target in future interventions have received less attention.

Although sleep problems are commonly reported among dementia caregivers, the nature and frequency of caregiver sleep disruptions and their relationship to health status have not been systematically mapped or summarized to inform future research (18,19). Thus, the objective of this review was to synthesize evidence regarding the link between caregiver burden and dementia caregivers’ sleep. Summarization of the findings was intended to provide a larger picture of sleep quality with caregiver burden and the impact of caregiver burden on an individual’s health.

## Method

The review was conducted according to the methodology of Arksey and O’Malley (20,21) following the stages of problem identification, literature search, study selection, data analysis, and presentation/discussion. This method was chosen because it allows for a broader understanding of the phenomena of sleep problems in dementia caregivers by including both experimental and nonexperimental research. The literature search was developed under the guidance of a professional medical librarian. Once the project team agreed on the keywords, the librarian performed the search in PubMed, CINAHL, and Web of Science in March 2022. The English language was the only search limit used across the databases. The PubMed search consisted of the MeSH terms and keywords listed below. Searches in the CINAHL and Web of Sciences databases were performed using keywords and phrases from the PubMed search: (“care partner” OR caregivers OR caregiver OR spouse OR family OR “adult child”) AND (dementia OR “vascular dementia” OR “Lewy body disease” OR “mixed dementia” OR “alzheimer disease” OR alzheimer OR “frontotemporal dementia”) AND (“care burden” OR “caregiver burden” OR stress OR stressor OR stressors OR burden OR “caregiver intensity” OR overload) AND sleep. After the initial search across the 3 databases, all results were exported into the citation manager, Sciwheel, where duplicates were removed. The remaining citations were exported into Covidence, a web-based collaboration software platform for literature reviews, to help manage the screening process.

### Title and Abstract Screening

All the articles were screened using the following inclusion criteria: (1) informal caregivers of persons living with dementia, (2) measured relationship between informal dementia caregiver sleep and subjective caregiver burden variables, and (3) original research (eg, not a protocol, review). Mild cognitive impairment was originally included as an inclusion criterion along with dementia; however, no manuscripts exclusively reported on this population. For this review, caregiver burden was operationalized as the perceived impact of caregiving involvement, as reported by the caregiver (22). Articles were only included if they specifically measured burden or stress deemed related to caregiving; articles that focused on depression or overall quality of life that were not measured by caregiving-specific instruments were not included. Non-English studies were excluded. All the articles were jointly assessed by 2 authors. Disagreements were resolved by the consensus of 3 researchers (ie, V.B., V.G., C.M., M.K.M., and K.M.S.).

### Full-Text Screening

Once title/abstract screening was completed, the full text of the articles was requested, and articles were jointly assessed by 2 authors. The final decision for inclusion was made by consensus (ie, V.B., V.G., C.M., M.K.M., and K.M.S.).

### Data Extraction and Analysis

We met the purpose of this review by systematically extracting predetermined data. After completing the final article list, team members (S.S., L.J., and M.K.M.) independently extracted data and created a table summarizing the findings for each article using the following headings: author, year; country; study aim; study design; sample and setting; care recipient cognitive impairment; measures of caregiver burden; measures of sleep; and primary outcome. Discrepancies were resolved by consensus among 2 team members who extracted the data. In alignment with the purpose of scoping reviews (23), the analysis was intentionally descriptive and intended to summarize the current literature on sleep and caregiver burden for dementia caregivers. In order to consider meaningful clinical implications of the findings, the team followed Levac et al. (24) recommendations to discuss implications for future research, practice, and policy.

## Results

**Figure 1.**
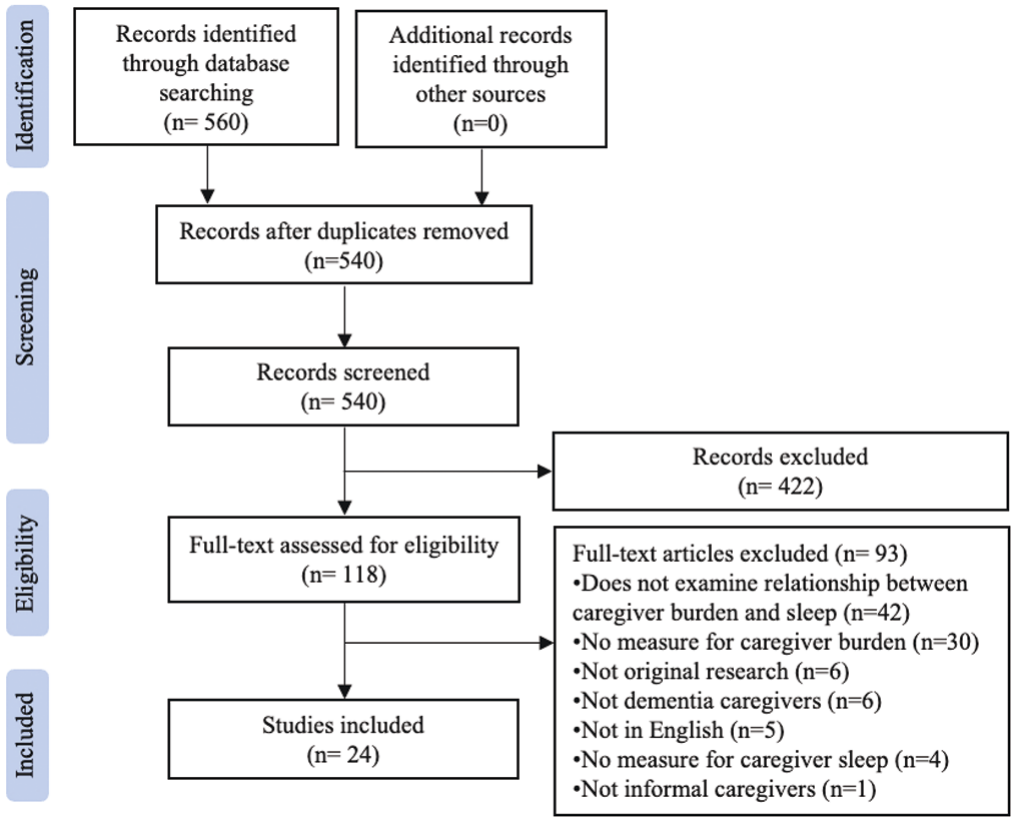
PRISMA flow diagram.

The literature search yielded 540 nonduplicate articles that were screened by title and abstract. After title and abstract screening, 118 full-text articles were reviewed. After reviewing the full-text articles, 24 studies were included that described the relationship between caregiver burden and sleep quality (see Figure 1).

Studies were conducted in 6 countries (USA, China, Taiwan, Italy, Canada, and Australia). The majority of the studies were cross-sectional with varying sample sizes ranging from 40 to 669 participants; 3 were longitudinal studies, each with more than 100 subjects; 1 was an experimental design with randomization of 450 participants; and 1 was a qualitative study with 14 participants. Seventeen studies included caregivers of persons with dementia; 6 studies included caregivers of people with AD; and 1 was composed of caregivers with AD, frontotemporal dementia, or Lewy body dementia. Supplemental Table 1 presents the subject characteristics, methods, and outcomes of the included studies.

### Measures of Caregiver Burden

Caregiver burden was measured most often with the Perceived Stress scale (*n* = 5). However, there was a significant variety in caregiver burden measurement across studies. Other measures included: Zarit Burden Inventory (*n* = 4), Caregiver Burden Inventory (*n* = 3), The Screen for Caregiver Burden (*n* = 2), Pearlin Stress Scale—Overload subscale (*n* = 2), Revised Memory and Behavior Problem Checklist (*n* = 1), Chinese Neuropsychiatric Inventory—Caregiver Distress scale (*n* = 1), Caregiver Burden scale (*n* = 1), Depression, Anxiety, and Stress scale-21 (*n* = 1), and Impact of Events scale (*n* = 1).

### Measures of Sleep

Included studies measured self-reported and/or objective sleep measures. Self-reported sleep variables were most often measured using questionnaires such as PSQI (*n* = 13), the Epworth Sleepiness Scale (*n* = 2), Sleep Hygiene Index (*n* = 1), Chinese General Sleep Disturbance scale (*n* = 1), Patient-Reported Outcomes Measurement Information System—Sleep Disturbance short form (*n* = 1), and Sleep Maintenance Insomnia (*n* = 1). Sensor-reported or objective sleep was also measured using actigraphy (*n* = 4) and polysomnography (*n* = 3).

### Comparison of Sleep Variables Between Caregivers and Noncaregivers

#### Self-reported metrics

Dementia caregivers had significantly poorer overall perceived sleep than the control group participants across 4 studies that compared self-reported sleep measures between caregivers and non-caregivers or healthy populations (25–28). However, even within studies, there were varied results when examining individual sleep instrument components (eg, sleep latency, sleep disturbances, and sleep duration of the PSQI) rather than the overall single instrument score. For example, Busse et al. (29) found that although caregivers’ PSQI sleep quality, habitual sleep efficiency, and daytime function were higher than nonpandemic normative data, there were no statistically significant differences (*p*s > .05) (29). One study (28) provided evidence of a statistically significant difference in overall PSQI sleep quality and individual components of sleep latency and sleep disturbance. However, the other individual PSQI components (sleep medications, sleep quality, sleep efficiency, daytime dysfunction, and sleep duration), were not statistically different between groups. Wilcox and King (28) also presented differences between caregivers and non-caregivers across overall PSQI and all individual PSQI components. However, no significant differences were noted between groups using multiple regression analysis.

#### Objective metrics

Two studies compared objective sleep quality using single sleep variables (eg, sleep latency) between caregivers and non-caregivers using polysomnography and found mixed results (25,30). Fonareva et al. (30) found that caregivers’ sleep onset latency and total sleep time in stage 1 of nonrapid eye movement sleep (NREM) were significantly longer for non-caregivers, while total sleep time in REM sleep stage was shorter than non-caregivers. Across all other polysomnography measures, there were no significant differences in sleep variables by group (30). Castro et al. (25) did not find significantly different sleep quantity or quality between caregivers and non-caregivers using polysomnography (30).

### Sleep Quality and Caregiver Burden

#### Self-reported metrics

Eighteen studies investigated the association between caregiver burden and self-reported sleep quality. Fourteen of these studies found a significant correlation between greater caregiving burden and worse sleep quality (26,31–43), whereas the remaining 4 studies showed that caregiver burden was not significantly correlated with subjective sleep quality measures (28,29,44,45). Of the 14 studies that measured overall sleep quality using the PSQI, 12 studies found a significant correlation between caregiving burden and overall sleep quality, while 2 did not (28,44).

#### Objective metrics

Four studies examined the relationship between caregiver burden and objectively measured sleep quality and showed varied results (33,36,41,46). Two of the 4 studies revealed that caregiver burden was not significantly correlated with any objective sleep quality measures collected (36,46). However, the other 2 studies found that caregiver burden was significantly associated with objectively collected sleep subdomain data. Sleep subdomain findings across the 4 articles provided evidence of a significant association between caregiver burden and increased sleep onset latency (*n* = 1, 100% of total times measured) (41); wake after sleep onset (*n* = 2, 100%) (36,41); sleep efficiency (*n* = 3, 33%) (36,41,46); and total sleep time (day and/or night; *n* = 3, 0% (36,41,46)).

#### Qualitative

One qualitative study was included and found that caregivers’ worry about their care recipients’ safety at night motivated caregivers to stay awake to listen for or get up and check on care recipients’ possible nighttime activity. Caregiver participants endorsed the feeling that nighttime supervision duties affect the quality and quantity of caregivers’ sleep (47).

## Discussion

This scoping review aimed to identify and synthesize evidence regarding the link between caregiver burden and dementia caregivers’ sleep. Dementia caregivers reported significantly poorer perceived overall sleep compared to their non-caregiver counterparts. However, differences between individual sleep subdomains were not consistently statistically significant between caregiver and non-caregiver groups. Overall sleep quality in dementia caregivers was significantly correlated with caregiver burden.

A recent meta-analysis by Gao et al. (17) found that caregivers of adults with AD experienced fewer hours of sleep and worse sleep efficiency than their noncaregiving counterparts. Similar to the articles included in Gao et al., our review identified the PSQI as the most common assessment of self-reported sleep quality. The overall PSQI score is composed of 7 subscores (subjective sleep quality, sleep latency, sleep duration, habitual sleep efficiency, sleep disturbances, use of sleep medication, and daytime dysfunction) (48) that were examined across most of the studies included in this review.

The PSQI, including both its overall score and subscores, provides an opportunity to understand sleep using a relatively fast, easy-to-administer, and validated instrument to inform interventions. Taking less than 10 minutes to complete, and based on known relationships between sleep measures and caregiver burden, it is reasonable to ask primary care providers to administer the PSQI or, at the very least, ask about sleep concerns that are more likely to occur in dementia caregivers compared to non-caregivers (eg, more frequent nighttime awakenings).

The findings from this review support Gao et al.’s recommendation to consider sleep quality subscores or subdomains when describing caregiver sleep (17). Although an overall sleep quality score, such as the PSQI, provides a general picture and severity rating, sleep interventions can or may be tailored to target specific subdomains (eg, a PSQI subscore endorsing the use of sleep medication may warrant a different approach). Contradictorily, using raw or subscale scores in analyses may increase the likelihood of dissimilar study findings due to the operationalization of sleep parameters using different scales. These dissimilar findings have the potential to contribute to inaccurate recommendations based on results derived from instruments assumed to be similar, but that used different scales during analysis.

Similar to our findings that different scales and subscales were used to measure sleep quality, there was also significant diversity across caregiver burden measures used in the included studies. As a complex and multifaceted issue (4), it was unsurprising that caregiver burden definitions and measures varied across studies. In this review, we operationalized caregiver burden as the caregiver-reported perceived impact of care involvement on their well-being. However, even when applying this definition, the review identified multiple relevant measures of caregiver burden. Although some of the caregiver burden scales in this review have subscales to represent different aspects of caregiver burden, most analyses used an overall caregiver burden score. As proposed previously with the sleep subscales, the caregiver burden subscales should be examined independently from the total score to identify the strength and direction of relationships between sleep quality and caregiver burden subdomains. For example, Creese et al. (49) found statistically significant relationships among different subdomains of sleep with caregiver subscales of role burden and personal burden. By isolating specific characteristics of caregiver burden related to sleep, and ideally certain aspects of sleep, there is an opportunity to identify caregiver-specific sleep concerns to target future interventions.

Most research used self-reported sleep metrics, and relatively fewer described the relationship between caregiver burden and objective sleep metrics (*n* = 4). Only 2 of these articles found an association between 4 sleep subdomains and caregiver burden, whereas more consistent associations were found between burden and self-reported sleep metrics. This may be due to the misalignment between objective sleep measures and how caregivers interpret or report their sleep. This is clinically relevant as the study findings from actigraphy did not overwhelmingly identify disrupted sleep, while self-reported measures from caregivers found more sleep concerns. The environment in which the data are collected (ie, home versus lab settings) may have also contributed to varied findings across the objective sleep measures. Although objective sleep measures may provide an opportunity to explore care partner sleep patterns, there remains a need for subjective accounts of sleep concerns, ideally by validated sleep subscores/domains, to allow for disentanglement from comorbid conditions. Based on varied findings using polysomnography and actigraphy, future work should consider collecting objective and subjective sleep measures concurrently as this may provide an opportunity to provide a comprehensive representative of sleep in real-time to aid in clinical diagnosis and treatment.

One of the aims of this review was to identify efficacious caregiver sleep interventions. Although 2 intervention studies were included in the review, only 1 was a randomized controlled trial, signaling a lack of experimental research to understand how improving sleep for caregivers of adults living with dementia may improve their caregiving burden (or vice versa). Although poor sleep quality is a known disparity, the reasons for worse sleep and modifiable factors related to sleep have received less attention when creating multimodal and holistic caregiver interventions. In a systematic review of sleep interventions for adult caregivers of any persons with care needs, 24 studies identified a variety of interventions that lacked long-term benefits (50). When findings are considered across adult caregivers, identifying the modifiable risk factors for poor sleep quality may provide a distinctive opportunity to improve caregiving health and well-being. For example, some of the interventions presented in the review and should be considered in future trials included cognitive-behavioral therapy for sleep, general caregiver health, exercise programs, acupressure, massage, reflexology, and music (50).

### Limitations

There were several limitations to this scoping review. All articles but 2 were identified as descriptive study designs, and there were limited sleep intervention studies that would have provided evidence of the direction of the relationship between sleep quality and caregiver burden. Based on the limited number of interventions identified in this review, we were unable to provide a summary of interventions for dementia caregivers designed to improve their sleep quality. Caregiver eligibility also varied across studies, which compounds concerns of generalizability across different caregiving populations. Review eligibility criteria ensured consistency in the target population of unpaid caregivers, irrespective of hours of care or relationship to the patient, which also presents diversity in the caregiver profile. Additionally, critical caregiver–recipient dyad considerations were not controlled for across the included studies. For example, not all studies collected data on the sleeping arrangements of the dyads (eg, shared beds, different rooms) or the amount of time caregiving, which may have provided additional risk factors affecting poor caregiver sleep quality. Overall, this review highlights the need for consistency when describing caregiver populations and measuring caregiver burden and sleep parameters in research.

## Conclusion

Caregivers of persons living with dementia have poorer self-reported sleep quality compared to their age-matched counterparts. This review found that although self-reported overall poorer sleep quality is associated with higher dementia caregiving burden, there is mixed evidence on the relationship between sleep quality subdomains and caregiver burden. Healthcare providers should consider the impact of care burden on caregivers’ sleep and regularly assess caregivers’ sleep difficulties. Future studies should focus on consistently measuring caregiver burden to promote dementia caregiver health and well-being through targeted interventions.

## Supplementary Material

igae005_suppl_Supplementary_Tables_S1
