# Supplementary material for: Sleep and Caregiver Burden Among Caregivers of Persons Living With Dementia: A Scoping Review
Source: Innov Aging. 2024 Feb 14;8(2):igae005. doi: 10.1093/geroni/igae005 (PMC10901478; doi:10.1093/geroni/igae005)
Supplement: igae005_suppl_Supplementary_Tables_S1 [file igae005_suppl_supplementary_tables_s1.docx]

*Innovation in Aging* Supplementary Material: Mattos et al. Sleep and caregiver burden among caregivers of persons living with dementia:

A scoping review.

Supplemental Table 1. Subject Characteristics, Methods, and Outcomes of Included Studies

| **Author**  **(year)** | **Country** | **Study Aim** | **Study Design** | **Sample and Setting** | **Cognitive Impairment** | **Measure(s) of Care Burden** | **Measure(s) of Sleep** | **Primary Outcome**  **(CG-non-CG comparison results are *italicized*)** | **Relationship between Caregiver Sleep and Burden** |
| --- | --- | --- | --- | --- | --- | --- | --- | --- | --- |
| Bussè et al.  (2022) ^29^ | Italy | 1. To assess the frequency and severity of long-term stress-related symptoms in CGs of persons living with dementia after one year of the pandemic.  2. To identify predictors of psychological outcomes. | Longitudinal | N = 101 informal CGs of persons living with dementia enrolled, 85 subjects with T1 and T2 evaluations included in analysis  N = 50, non-CGs | Dementia | CBI | PSQI | *CGs’ PSQI sleep quality, habitual sleep efficiency, and daytime function were higher than non-pandemic normative data of whole population, but not significant (ps>.05).*  *CGs showed more use of sleeping medication than non-CGs (p=.003).* | *CG subgroup only (n=85)  -PSQI-sleep quality & CBI-developmental burden, emotional burden (+)  -PSQI-sleep quality & CBI time developmental burden (-)  -PSQI-sleep efficiency & CBI-emotional burden (+)  -PSQI-sleep efficiency & CBI-time developmental burden, developmental burden (-)  -PSQI-sleep meds & CBI-developmental burden, time developmental burden, and emotional burden (-)  -PSQI-daytime dysfunction & CBI-developmental burden (+)  -PSQI-daytime dysfunction & CBI-time developmental burden and emotional burden (-) |
| Castro et al. (2009)^25^ | USA | 1. To describe subjective and objective sleep characteristics in women who are dementia family CGs compared to women who are non-CGs.  2. To examine actigraphy data on the care recipients to explore the correspondence of the care recipients’ sleep-wake patterns in relation to the CGs. | Cross-sectional | N = 6, women family CGs for a spouse or parent with dementia  N = 34, comparable non-caregiving women | Dementia | SCB | PSG, PSQI, ESS | *CGs and non-CGs did not have significantly different sleep quantity and quality results on any PSG measures.*  *In the older age group (ages 65+ years old), there were differences in PSQI sleep latency and sleep disturbances between CGs and non-CGs (p < 0.05). PSQI global, PSQI perceived hours sleep, PSQI daytime dysfunction, PSQI sleep latency, PSQI sleep duration, PSQI subjective sleep quality, and PSQI sleep medications were not significantly different from non-CG.*  *PSG sleep variables were not associated with CG burden (ps > .05).* | PSG  -TST (-)  -Sleep efficiency (-)  -Sleep latency (-)  -WASO (-)  -Time in Stage 1 (-)  -Time in Stage 2 (-)  -Time in SWS (-)  -Time in REM (-)  -Awakenings per night (-)  -PLMS per hour (-)  *relationship between SCB and ESS or PSQI not reported, collected as descriptive variables |
| Chiu et al. (2014)^31^ | Taiwan | To explore the relationships of family CGs’ sleep disturbance with multilevel stressors. | Cross-sectional | N = 180, family CGs who provided all or most assistance to persons living with dementia for the past 3 months, or who provide secondary care to their relative by supervising a hired care assistant | Dementia | CNPI-Caregiver Distress Scale | Chinese GSDS | There were positive associations between family caregiving distress and most sleep disturbance domains (difficulty falling asleep, waking up during sleep, waking up before the end of a sleep cycle, sleep quantity, dozing in the daytime, and consumption of sleep pills; ps<.05).  Sleep quality domain and CG distress did not demonstrate a significant correlation (p=.07).  Hierarchical model analysis showed about 30% of the variance in sleep disturbance can be explained by caregiving distress and fatigue. | GSDS (+)  -Difficulty falling asleep (+)  -Waking up during sleep (+)  -Waking up before the end of a sleep cycle (+)  -Sleep quantity (+)  -Dozing in the daytime (+)  -Consumption of sleep pills (+)  -Sleep quality (-) |
| Coffman et al. (2017)^26^ | USA | To explore the demographic characteristics of this technology-enabled AD CG sample, as well as cross-sectional relationships among CG burden and several mental health measures. | Cross-sectional | N = 165, AD CGs between the ages of 45–75 years who reported using a smartphone and who had Internet access | AD | ZBI | PROMIS-SD Short Form | *Approximately 25% of the CGs scored greater than one standard deviation above the population average, suggesting relatively poor sleep quality.*  CG burden and sleep disturbance were positively correlated (p < 0.001). | PROMIS sleep disturbance (+) |
| Cothran et al. (2022)^32^ | USA | 1. To describe subjective stress and objective stress in African American family CGs.  2. To explore relationships between CG demographics, subjective stress, objective stress, social determinants of health, and health outcomes. | Cross-sectional (secondary data analysis) | N = 142, family member (aged 21+) with AD, providing at least four hours of daily care in a home setting | AD | PSS  *ZBI collected as a descriptive variable | PSQI | Perceived stress of CGs was significantly associated with sleep quality (p < 0.001). | Global PSQI (total score, +) |
| Creese et al. (2008)^49^ | Canada | 1. To investigate the sleep characteristics of a sample of spousal CGs currently residing with the person living with dementia. | Cross-sectional | N = 60, spousal CGs of individuals with AD | AD | A short version of ZBI | Five-item scale (items 1,3, and 5 from PSQI) | CG sleep quality, change in sleep quality, and frequency of nocturnal disruptions by their spouse were all positively correlated with CG role burden (ps = 0.001).  CG sleep quality, change in sleep quality, and frequency of nocturnal disruptions by their spouse were not correlated with CG personal burden. | Modified PSQI & CG role burden  -Sleep quality (+)  -Change in sleep quality (+)  -Freq of nocturnal disruptions (+)  Modified PSQI & CG personal burden  -Sleep quality (-)  -Change in sleep quality (-)  -Freq of nocturnal disruptions (-) |
| Elliott et al. (2010)^38^ | USA | 1. To examine the relationships between changes from baseline to after the intervention in CG self-reported health, caregiving burden, and caregiving bother. | RCT | N = 450, dementia CG and CR dyads who participated in the REACH II study | Dementia | ZBI  REACH II subscale measuring frustrations with caregiving | A sub-item of REACH II measuring “overall sleep quality” (p.4) | CG sleep quality was significantly correlated with frustrations with caregiving (p =< .05), emotional burden (r = -0.23, p = 0.001), and role burden (r = -0.18, p = .001). | “Overall sleep quality” (+) |
| Fonareva et al. (2011)^30^ | USA | 1. To evaluate sleep in dementia CGs using a comprehensive sleep assessment utilizing an ambulatory polysomnography device. | Cross-sectional | N = 20, primary CGs for a relative with dementia  N = 20, non-CGs | Dementia | PSS  RMBPC as a descriptor only | PSQI, ESS, PSG | *There were no significant differences between CG and non-CG groups on ESS (p = .32), but there was a significant difference in PSQI for the CG group compared to the non-CG group (p < .001).*  *CGs’ SOL and TST in NREM stage N1 were significantly longer than non-CGs’ (p = 0.39). CGs’ TST in the REM stage was shorter than non-CGs’ (p = 0.029).* | *Global PSQI (+)*  *ESS (-)*  *PSG*  *-% TST in stage N2 (-)*  *-% TST in stage N3 (-)*  *-% TST in stage R (+)*  *-% TST in N1 (+)*  *-SOL (+)*  *-Time in bed (-)*  *-WASO (-)*  *-Arousals during sleep (-)*  *-Sleep efficiency (-)* |
| Glover et al. (2006)^46^  Conference abstract | USA | 1. To describe the sleep patterns of persons living with dementia.  2. To explain the relationship between CG burden and total nighttime awakenings, TST, and sleep efficiency in persons living with dementia | Cross-sectional (secondary data analysis) | N = 42 dyads comprised of  persons living with dementia and their CGs who reported five or more nighttime behaviors at least three times a week | Dementia | CBS | PSG | CG burden was not significantly correlated with the sleep variables of interest. | PSG  Sleep efficiency (-)  TST (-)  Total nighttime awakenings (-) |
| Kim (2015)^39^  Conference abstract | USA | To examine the effect of caregiving stress on depression among elderly spouse CGs of AD patients. | Cross-sectional (secondary data analysis) | N = 267, spouse CGs as a subset of the REACH II study | Dementia | Unknown (“caregiving stress”) | unknown (“sleep quality”) | CGs’ sleep quality partially mediates the impact of caregiving stress on depression. | Unknown measure  Sleep quality (+) |
| Liang et al. (2020)^40^ | USA | 1. To explore the association between role overload and sleep maintenance insomnia, and the moderation effects of social support and social engagement.  2. To explore the mediating effect of sleep quality and social support on the relationship between caregiving stress and depression. | Cross-sectional (secondary data analysis) | N = 669, CGs who participated in the 2015 NSOC | Possible or probable dementia^51^ | Pearlin Stress Scale- Overload subscale | SMI: Single-item: “In the last month, on nights when you woke up before you wanted to, how often did you have trouble falling back asleep?” | Role overload of dementia CG was positively associated with the risk of SMI (p < 0.01).  Differences between adult children and spousal caregivers:  - Only adult children caregivers’ perceived role overload is positively associated with their SMI (β = .147 (.022) when the moderation effect is not considered. | SMI (+) |
| Liu et al. (2017)^44^ | China | To evaluate CG burden and factors that influence this burden among CGs and patients with AD. | Cross-sectional | N = 309 dyads, CGs, and the patient with dementia from a hospital neurology clinic | Dementia or probable/  possible AD^51^ | ZBI | PSQI | The relationship between CGs’ PSQI scores and ZBI scores was not statistically significant. | Global PSQI (-) |
| Oken et al. (2011)^27^ | USA | To evaluate whether the stress of being the primary CG of persons living with dementia produces cognitive dysfunction. | Cross-sectional | N = 31, CGs of persons living with dementia  N = 25, non-CGs  Non-CGs were matched on the age and gender of CGs of persons living with dementia | Dementia | PSS | PSQI | There were statistically significant differences in PSQI and PSS scores, between CGs and non-CGs (ps = 0.0001, 0.0016).  Zero-order correlation between PSS and PSQI for CG v. controls (r=.419, p<.005) | Global PSQI (+) |
| Osakwe et al. (2022)^45^ | USA | To examine racial/ethnic differences in sleep disturbance and strain among family CGs of persons living with dementia | Cross-sectional | N = 1142, CGs and persons living with dementia from the 2017 NHATS and NSOC | Dementia, and possible or probable dementia^51^ | Six-item scale measuring CG strain | Two-item scale assessing CG sleep-related disturbances: 1) How often is sleep interrupted and how often is trouble failing back asleep?”  2) “In the  last month, on nights when you woke up before you wanted  to, how often did you have trouble falling back asleep?” | A higher frequency of assistance with personal care was associated with greater sleep interruption.  No association between a high level of CG strain and sleep initiation or sleep maintenance (ps>.09). | Sleep maintenance (-)  Sleep initiation (-) |
| Peng, Chang, and Wray (2012)^33^  Conference abstract | USA | To examine the effects of depression and burden on dementia CGs’ sleep. | Cross-sectional | N = unknown, the study still recruiting, family CGs | Dementia | CBI | Actigraphy, PSQI | Preliminary findings indicate that CG burden is associated with waking up during the night (r = 0.40, p < 0.05) and awake times (r = 0.43, p < 0.05) measured by Actigraphy.  CG burden is associated with PSQI global score (r = 68, p < 0.001), sleep disturbance (r = 64, p < 0.001), and sleep latency (r = 0.60, p < 0.001). | Actigraphy  Night awakenings (+)  Wake times (+)  Global PSQI (+)  Sleep disturbance (+)  Sleep latency (+) |
| Peng, Lorenz, and Chang (2019)^41^ | USA | To examine the association among CGs’ depression, burden, health status, sleep hygiene, care recipients’ sleep, and objectively measured CGs’ sleep. | Cross-sectional | N = 43, family CGs of persons living with dementia | Dementia | CBI | PSQI, SHI, Sleep diary, Actigraphy | CG burden significantly predicted their wake time after sleep onset (t = 2.311, p < 0.05). CGs with high levels of burden had longer sleep latency (r = 0.35, p < 0.05), lower sleep efficiency (r = -0.40, p < 0.01), and awoke more frequently after sleep onset (r = 0.36, p < 0.05).  CG burden was significantly correlated with PSQI global score (r = 0.51, p < 0.01), daytime dysfunction (r = 0.52, p < 0.01), sleep disturbances (r = 0.45, p < 0.01), sleep efficiency (r = 0.27, p < 0.05), and sleep duration (r = 0.29, p < 0.05). | Actigraphy  WASO (+)  Sleep latency (+)  Sleep efficiency (+)  TST (-)  Awake time during the night (-)  Global PSQI score (+)  Daytime dysfunction (+)  Sleep disturbances (+)  Sleep efficiency (+)  Sleep duration(+)  SOL (-) |
| Polenick et al. (2018)^43^ | USA | To evaluate the association between CBs’ medical/nursing tasks and care-related sleep disturbances. | Cross-sectional | N = 104, spousal CGs and persons living with dementia | Dementia | Role overload, three Likert-scale items | One item asked about the frequency of sleep interruption in the last month | Bivariate association between sleep disturbance and role overload (r=.20, p=.05).  CG role overload was not significantly associated with sleep disturbances in hierarchical regressions. | Sleep disturbances (+, p=0.05) |
| Smyth et al. (2020)^34^ | Australia | 1. To investigate the sleep characteristics and disturbances of CGs of persons living with dementia.  2. To evaluate the associations between mood and sleep.  3. To identify significant predictors of poor sleep in CGs of persons living with dementia. | Cross-sectional | N = 104, informal CGs of persons living with dementia | Dementia | DASS-21 | PSQI | CG stress was significantly correlated with PSQI global score (r = 0.41, p < 0.001).  In forward stepwise regression analysis, stress scores were significantly associated with PSQI global scores (β = 1.27, t = 3.553, p = 0.001). | Global PSQI score (+)  Sleep quality (+)  Sleep latency (+)  Sleep disturbances (+)  Use of sleeping medication (+)  Daytime dysfunction (+)  Sleep duration (-)  Sleep efficacy (-) |
| Spring et al. (2009)^47^ | USA | 1. To address what psychological, sleep, or other problems are associated with providing nighttime supervision to persons living with dementia.  2. To explore the CG benefits of using the NMS, and how those benefits are conveyed. | Qualitative | N = 14, CGs of persons living with dementia | Dementia | n/a | n/a | Consequences of nighttime supervisory role:  -increase in CG worry  -causes disruption in CG sleep, both quality and quantity  -loss of personal space or privacy  Poor sleep as a result of worry included additional consequences:  -decreased energy  -changes in mood | Sleep quality and quantity (+) |
| von Känel et al. (2014)^35^ | USA | To examine the longitudinal relationship between positive affect and sleep. | Longitudinal | N = 126, spousal CGs | AD | Four-item scale assessing role overload with life responsibilities | PSQI, Actigraphy | CG role overload was significantly associated with PSQI scores (p < 0.001). | Global PSQI (+)  Actigraphy not reported |
| von Känel et al (2012)^36^ | USA | To examine the longitudinal effects of dementia caregiving and major transitions in the caregiving situation on CGs’ sleep and the effect of moderating variables. | Longitudinal | N = 109, spousal CGs of people with AD  N = 48, non-CG controls | AD | Pearlin Stress Scale- Overload subscale | PSQI, Actigraphy | CG role overload was significantly associated with PSQI scores (p < 0.001). However, CG role overload had no significant relationship with objective sleep measures by actigraphy. | Global PSQI (+)  Actigraphy  Nighttime TST (-)  Nighttime WASO (-)  Nighttime sleep percent (-)  Daytime TST (-) |
| Wang et al. (2016)^37^ | Taiwan | 1. To explore the relationship between perceived stress and depressive symptoms.  2. To examine whether caregiving self-efficacy and sleep disturbance are relevant constructs in contributing to this relationship among CGs. | Cross-sectional | N = 72, family CGs for patients with AD | AD | PSS, RMBPC-reaction | CPSQI | CG perceived stress (r = .28, p < 0.05) and RMBPC (r = .27, p < 0.05) were significantly correlated to global PSQI scores. | Global PSQI (+) |
| Wilcox & King (1999)^28^ | USA | 1. To examine the prevalence of sleep complaints among female family CGs for persons living with dementia.  2. To evaluate whether sleep complaints vary as a function of CG relationship or CR diagnosis.  3. To describe the nature and correlates of their sleep problems. | Cross-sectional | N = 90, women CGs for persons living with dementia | Dementia | SCB, PSS | PSQI | *CGs were significantly more impaired than healthy adults on the PSQI components of sleep quality, sleep latency, sleep duration, sleep efficiency, sleep medication, sleep disturbances, daytime dysfunction, and PSQI global sleep score (all ps < .001).*  *In multiple regression analysis, CG burden was not significantly related to PSQI global scores and PSQI components (ps>.05).* | *Global PSQI (+) when comparing CGs vs. non-CGs differences*  *However, multiple regression analysis that controlled for covariates showed there was not a significant relationship for:*  *Global PSQI (-)*  *Sleep quality (-)*  *Sleep latency (-)*  *Sleep duration (-)*  *Sleep efficiency (-)*  *Sleep medication (-)*  *Sleep disturbances (-)*  *Daytime dysfunction (-)* |
| Wilson et al. (2018)^42^ | USA | To examine dementia family CGs’ risks for heightened pain, sleep problems, and depressive symptoms, in relation to the cycles of intrusive thoughts and avoidance that characterize caregiving-related distress. | Cross-sectional | N =72, spousal CGs of persons living with dementia  N = 58, adult children CGs of persons living with dementia | Dementia | IES | PSQI | CGs with more CG-related distress had significantly more sleep problems than CGs with lower distress (p < .0001). | Global PSQI (+) |

*Note*. * = *Italicized* words/sections denote sleep quality comparison between caregiver and non-caregiver samples; National Health and Aging Trends Study (NHATS) criteria was used to identify participants with probable or possible dementia;^51^ AD = Alzheimer’s Disease; CBI = Caregiver Burden Inventory; CBS = Caregiver Burden Scale; CG = caregiver; CR = care receiver; CNPI = The Chinese Neuropsychiatric Inventory; DASS = Depression, Anxiety, and Stress Scale; GSDS = General Sleep Disturbance Scale; ESS = The Epworth Sleepiness Scale; FLD = frontotemporal lobar degeneration; IES = Impact of Events Scale; ISI = Insomnia Severity Index; LB = Lewy bodies; NHATS = National Health and Aging Trends; NMS = nighttime monitoring system; non-CGs= non-caregivers; NSOC = National Study of Caregiving; PSG = polysomnography; SCB = The Screen for Caregiver Burden; SHI = Sleep Hygiene Index; SMI = Sleep Maintenance Insomnia; PD = Parkinson’s disease; PROMIS-SD = Patient-Reported Outcomes Measurement Information System - Sleep Disturbance; PSS = Perceived Stress Scale; PSQI = Pittsburgh Sleep Quality Index; RCT = Randomized clinical trial; REACH = the Resources for Enhancing Alzheimer’s Disease Caregiver Health II; RMBPC = Revised Memory and Behavior Problem Checklist; RU SATED = regularity satisfaction alertness timing efficiency duration; SOL = sleep onset latency; TST = Total sleep time; WASO = wake after sleep onset; ZBI = Zarit Burden Interview
